# Health care consumption and psychiatric diagnoses among adolescent girls 1 and 2 years after a first-time registered child sexual abuse experience: a cohort study in the Stockholm Region

**DOI:** 10.1007/s00787-020-01670-w

**Published:** 2020-11-01

**Authors:** Gita Rajan, Sanna Syding, Gunnar Ljunggren, Per Wändell, Lars Wahlström, Björn Philips, Carl Göran Svedin, Axel C. Carlsson

**Affiliations:** 1 Division for Family and Community Medicine, Department of Neurobiology, Care Sciences and Society, Karolinska Institutet, Alfred Nobels Allé 12, 141 83 Huddinge, Sweden; 2 Academic Primary Healthcare Centre, Stockholm, Sweden; 3 Centre for Psychiatry Research, Karolinska Institutet, Stockholm, Sweden; 4 Department of Social Sciences, Ersta Sköndal Bräcke University Collage, Stockholm, Sweden; 5 Department of Psychology, Stockholm University, Stockholm, Sweden

**Keywords:** Sexual abuse, Administrative databases, Comorbidity, Medication, Epidemiology

## Abstract

Child sexual abuse (CSA) is a crime against human rights with severe health consequences, and suicidal actions, stress, eating disorders, and borderline disorder are common among survivors of CSA. The objective of this study was to analyze how health care consumption patterns developed among adolescent girls in the Stockholm Region, Sweden, 1 and 2 years after the first registration of CSA experience appeared in their medical record, as compared to age-matched controls without such registration. In this cohort study, number of healthcare visits, comorbidities, and prescribed drugs were collected through the Stockholm Region administrative database (VAL), for girls age 12–17 with registration of CSA experience in their medical record (*n* = 519) and age-matched controls (*n* = 4920) between 2011 and 2018. Healthcare consumption patterns remained higher among the girls with a registered CSA experience compared to the controls, both 1 and 2 years after the first CSA experience registration. Highest odds ratios (ORs) were found for suicide attempts [OR 26.38 (12.65–55.02) and 6.93 (3.48–13.49)]; stress disorders [25.97 (17.42–38.69) and 15.63 (9.82–24.88)]; psychosis [OR 19.39 (1.75–214.13) and 9.70 (1.36–68.95)], and alcohol abuse [OR 10.32 (6.48–16.44) and 6.09 (1.98–18.67)], 1 and 2 years, respectively, after the first CSA experience registration. The drug prescriptions were also significantly higher among the girls with a CSA experience registration than for the controls. The results highlight the need to systematically evaluate and develop assessment, treatment planning, and interventions offered to adolescent girls after their first CSA experience registration.

## Introduction

Sexual abuse is a crime with a high prevalence all over the world, with often devastating health consequences. A meta-analysis of 331 international studies from 2011 showed an overall estimated prevalence of self-reported child sexual abuse (CSA) to be 12.7% (girls 18.0% and boys 7.6%) [1]. Several international surveys and interview studies have shown that the risk of mental illness and substance abuse, as well as physical illness and somatic pain, is more common after interpersonal traumas among both females and males, especially when they have occurred during childhood [2–16]. New technique and electronic journal records have opened up new possibilities for analyzing healthcare data. In 2017, we replicated prior results, and quantified with real numbers by a register-based study including over 2 million persons in Stockholm Region, Sweden [17]. The odds for stress-related diagnoses and depression were tenfold higher among those with a sexual abuse (SA) experience registration, compared to those without SA experience registration [17]. However, in that study, the comorbidities and the registered SA experience were collected in the same time frame. In 2018, medical electronic records for female adult patients with diagnoses of sexual abuse (SA) at the Kaiser Permanente Northern California were analyzed and compared to matched controls 1 year before and after the diagnoses of SA were recorded [18]. High healthcare consumption as well as a high level of comorbidities were found both before and after the diagnoses of SA were recorded [18]. In 2019, we presented a second study using the register-based health care data from the Stockholm Region. In this case–control study, we analyzed the healthcare consumption patterns of the sexually abused adolescent girls, 2 and 1 years prior to their first registered CSA experience, and compared them to matched controls [19]. The health care consumption among the girls with a registered CSA experience was drastically higher, but also differed remarkably from their matched controls. For 64% of the adolescent girls with a later registered CSA experience, the registration was made at the emergency ward for rape victims [19]. One conclusion from the study was that with proper knowledge, these girls could have been identified and offered preventive interventions already 2 years before the first registration of the CSA. The aim of this study was to follow the same cohort and analyze how their healthcare consumption levels and patterns developed, including whether there were different developmental trajectories regarding the various diagnoses, 1 and 2 years after the first registered CSA experience. Because of the low prevalence of boys with CSA experience registration in the original cohort (*n* = 19), only girls were included in the study.

## Methods

Stockholm Region has today over 2.2 million inhabitants, representing more than one-fifth of Sweden’s entire population. The region includes the capital city of Stockholm and several other cities and towns, as well as large rural areas and a sparsely populated archipelago. The Stockholm Region is responsible for financing primary and secondary health care, mainly through taxes. With the exception of very few private clinics that operate without subsidies in Stockholm, all consultations and diagnoses are recorded and stored in a group of databases, the Stockholm Regional Health Care Data Warehouse (VAL). These databases compile and store data on health care utilization from primary care, specialist open care, hospital inpatient care, and data on collected prescribed medications [20, 21]. The use of VAL makes it possible to perform prevalence and incidence studies for different diagnoses for all residents (and temporary visitors). As an indication for its accuracy and validity, VAL is used by the Council for updating the National Patient Register kept by the Swedish National Board of Health and Welfare (NBHW) as well as the annual benchmarking reports of the NBHW and the Swedish Association of Local Authorities and Regions (SKR) [22]. Since 1997, diagnoses have been coded according to WHO’s International Classification of Diseases, 10th edition (ICD-10).

### Study population

The studied cohort in the present study was defined as all living girls, age 12–17, who resided in Stockholm Region at some point between 1 January 2011 and 31 December 2018. Data on all health care consultations in primary care and specialized open care during 2011–2018 were extracted from VAL. Girls with a disclosed CSA experience registered in their medical records were used as cases (*n* = 519). Approximately ten controls without registered CSA experiences matched for age and socio-economic, per girl with a registered CSA experience, were used to compare their consumption of health care. Each control was only enrolled once, even if she matched more than one of the cases. Therefore, the number of controls (*n* = 4920) is less than ten times the number of cases (*n* = 519).

### Socio-demography

We used the Mosaic tool as classification of neighborhood socio-economic status into three levels, i.e., high, middle, or low. Mosaic is a tool developed by the marketing company Experian, to classify consumers to make sale activities more effective. The Mosaic system makes it possible to achieve a nuanced classification of socio-economic status. It uses a multivariate modeling utilizing over 400 variables to group postcodes into different types and aggregated broader groups. It uses data from 29 different countries, and has been shown to be useful also for the classification of cohorts in epidemiologic research [21, 23].

### Design

This was a cohort study, comparing health consumption patterns among girls, age 12–17, with a CSA experience registration with controls without such experience registered. Diagnoses and registration of CSA experience were registered after a consultation with a doctor, psychologist, or therapist. The data give no information about when the abuse occurred, only when it was disclosed and registered into the medical record. The healthcare data were collected at two times, 1 and 2 years (1–365 days and 366–730 days) after the first registered CSA experience. The data were also compared with the data from the earlier study [19] that measured health care data 1 and 2 years (1–365 days and 366–730 days) prior to the first registered CSA experience. The following ICD codes were used to define CSA experience registration: Consultation and observation after a reported rape Z04.4; sexual abuse by person without weapon Y05; child sexual abuse, by parent; Y07.1C child sexual abuse by other than parent; Y07.8C; sexual abuse T74.2.

Relevant co-morbid diagnoses were chosen from an earlier study on the same population [19]: Reactions of stress F43; anxiety disorders F40, F41; psychotic disorders F20, F23, F25, F28, and F29; bipolar disorders F30 and F31; alcohol abuse F10; other substance abuse F11–F16, F18–F19; depression F32, F33; pain from stomach, head, muscles, and joints R10, R51, R52, G44, and M79; ADHD F90; autism spectrum disorder F84; borderline personality disorder F60.3; eating disorder F50 and self-harm behavior X60-84. Pharmaceutical codes (ATC) were selected to mirror sleep disturbances and psychiatric ails: tranquilizers (R06AD02, N05BB); neuroleptics (N05A); propiomazine (N05C, M06); antidepressant drugs (N06A); melatonin (N05CH01); hypnotics (N05CF); stimulants (N06BA); and benzodiazepines (N05CD, N05BA).

### Ethics

All data were anonymized and none of the individuals could be identified. Management and analysis based on VAL is part of a continuous quality control of health care utilization in Stockholm Region. Ethical approval has been obtained from the Regional ethical review board in Stockholm to study diseases and their comorbidities with these data (permits: 2013/2196-31/2, 2016/638-32).

### Statistical methods

Means and 95% confidence intervals (CI) were calculated to compare number of health care visits between girls with a registered CSA experience and their controls. Conditional logistic regression was used to calculate odds ratios (ORs) with 95% confidence intervals (CIs), to compare the odds of concomitant disorders and prescribed drugs between the girls with a registered CSA experience and their controls. Due to the matching of age and socio-economic status, we did not adjust our data further. Statistical analysis and data management were performed using SAS software, version 9.4 (SAS Institute Inc., Cary, NC).

## Results

**Fig. 1:**
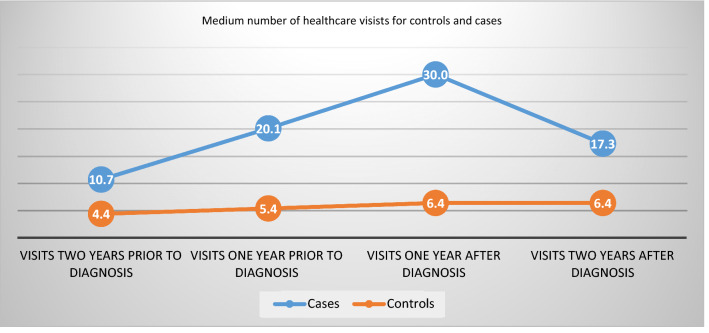
Numbers of healthcare visits per year, among adolescent girls with a registered CSA experience (cases), as compared to adolescent girls without a registered CSA experience (controls), 1 and 2 years prior and after the first registered CSA experience. The numbers prior to the first registered CSA experience are collected from a previous study in the same cohort [19]

The number of health care visits, planned and completed, in total and in different clinics, is presented in Table 1. In Fig. 1, the total numbers of visits 1 and 2 years after the first registered CSA experience are supplemented with the numbers of visits 1 and 2 years *prior* to the first registered CSA experience from a previous study on the same cohort [19]. The total number of visits in Table 1 (including no shows) was higher for the girls with CSA experience registration at all time points, with the highest difference 1 year after the first registered CSA experience. The mean number of visits for these girls 1 year after the first registered CSA experience was 30.03 (27.71–32.34) and 6.37 (6.01–6.73) for the controls. The most frequent place to visit healthcare the first year after the first registered CSA experience was to outpatient clinics, 28.20 (26.09–30.31) for girls with a CSA experience registration and 5.93 (5.58–6.27) for the controls. The least common type of visit 1 and 2 years after the first registered CSA experience was to the emergency ward 1.83 (1.21–2.45) and 0.44 (0.40–0.49) for girls with a CSA experience registration and controls, respectively.

**Table 1:** Number of health care visits per year, in adolescent girls with a CSA experience registration and their matched controls 1 (1–365 days) and 2 years (366–730 days) after the first registered CSA experience (2011–2018)

Clinics visited	Cases 1 year after diagnosis *n* = 519 (CI 95%)	Controls 1 year after diagnosis *n* = 4920 (CI 95%)	Cases 2 years after diagnosis *n* = 519 (CI 95%)	Controls 2 years after diagnosis *n* = 4920 (CI 95%)
All clinic visits	30.03 (27.71–32.34)	6.37 (6.01–6.73)	17.31 (15.56–19.07)	6.41 (6.09–6.73)
Emergency clinic visits	1.83 (1.21–2.45)	0.44 (0.40–0.49)	1.22 (0.91–1.53)	0.44 (0.40–0.48)
Outdoor clinic visits	28.20 (26.09–30.31)	5.93 (5.58–6.27)	16.09 (14.48–17.71)	5.97 (5.66–6.28)
Psychiatry clinic	20.80 (18.67–22.93)	2.0 (1.77–2.22)	10.45 (8.90–12.00)	2.24 (2.00–2.45)
Primary health care	4.08 (3.59–4.58)	2.62 (2.39–2.85)	3.55 (3.09–4.00)	2.35 (2.22–2.48)
Other specialist clinic	3.11 (2.83–3.40)	0.65 (0.60–0.69)	1.53 (1.26–1.80)	0.75 (0.69–0.81
Pediatric clinic	2.03 (1.75–2.31)	1.12 (1.03–1.18)	1.79 (1.51–2.07)	1.06 (1.00–1.13)
“No show” at planned visits	3.08 (2.74–3.43)	0.34 (0.30–0.38)	1.98 (1.67–2.29)	0.37 (0.33–0.41)
Professionals visited				
Psychologist, therapist, social worker	15.16 (13.66–16.66)	1.47 (1.31–1.63)	5.94 (4.94–6.94)	1.46 (1.30–1.61)
Physician	7.74 (7.08–8.41)	2.63 (2.53–2.73)	5.70 (5.12–6.23)	2.59 2.48–2.70)
Other	7.12 (6.16–8.09)	2.27 (2.037–2.50)	5.67 (4.88–6.46)	2.36 (2.21–2.52)

The odds ratios (ORs) of concomitant disorders for the girls with a CSA experience registration and the controls are presented in Table 2. The girls with a CSA experience registration showed significantly higher ORs for concomitant disorders than the controls at all time points. The highest ORs were for suicide attempts; OR 26.38 (12.65–55.02) 1 year and OR 6.93 (3.48–13.49) 2 years after the first registered CSA experience; stress 25.97 (17.42–38.69) 1 year and 15.63 (9.82–24.88) 2 years after the first registered CSA experience; psychotic disorders OR 19.39 (1.75–214.13) 1 year and 9.70 (1.36–68.95) 2 years after the first registered CSA experience; alcohol abuse OR 10.32.

**Table 2:** Odds ratios for relevant comorbidities and drug prescriptions of relevant pharmacotherapies in girls with a CSA experience registration compared to controls 1 (1–365 days) and 2 years (366–730 days) after the first registered CSA experience (2011–2018)

Diagnoses	OR 1 year after diagnosis *n* = 519 (CI 95%)	OR 2 years after diagnosis *n* = 519 (CI 95%)
Psychosis (F20, F23, F25, F28, F29)	19.39 (1.75–214.13)	9.0 (1.36–68.95)
Suicide attempt (× 6)	26.38 (12.65–55.02)	6.93 (3.48–13.49)
Stress (F43)	25.97 (17.42–38.69)	15.63 (9.82–24.88)
Alcohol abuse (F10)	10.32 (6.48–16.44)	6.40 (3.88–10.55)
Depression (F32, F33)	4.67 (3.30–6.60)	3.02 (2.08–4.39)
Anxiety (F 40, F41)	4.59 (3.45–6.09)	3.46 (2.54–4.72)
Autism (F.84)	3.18 (1.86–5.46)	3.72 (2.15–6.44)
ADHD (F90)	3.85 (2.86–5.18)	3.65 (2.70–4.93)
Pain (R10, R51, R52, G44, M79)	1.63 (1.28–2.07)	1.55 (1.21–1.99)
Eating disorders (F50)	1.66 (0.70–3.97)	2.05 (0.95–4.42)
Bipolar disorder (F30, F31)	3.23 (0.65–16.05)	2.65 (0.74–9.51)
Borderline (F60)	2.42 (0.27–21.67)	6.09 (1.98–18.67)
Pharmaceutical drug type		
Tranquilizers (R06AD02, N05BB)	8.37 (6.58–10.64)	5.81 (4.40–7.66)
NRLP (N05A)	10.92 (6.47–18.43)	8.93 (5.38–14.84)
Propiomazine (N05CM06)	7.55 (4.63–12.32)	8.11 (5.22–12.61)
Antidepressant drugs (N06A)	7.34 (5.79–9.31)	5.51 (4.30–7.05)
Melatonin (N05CH01)	6.26 (4.88–8.05)	4.48 (3.33–6.03)
Hypnotics (N05CF)	9.88 (4.56–21.43)	3.40 (1.51–7.64)
Stimulants (N06BA)	4.13 (3.12–5.47)	4.67 (3.54–6.17)
Benzodiazepines (N05CD, N05BA)	3.38 (1.64–6.97)	2.16 (0.89–5.26)

(6.48–16.44) 1 and 6.40 (3.88–10.55) 2 years after the first registered CSA experience.

The ORs of dispensed drugs between the girls with a CSA experience registration and the controls are presented in Table 2. The girls with a CSA experience registration had higher ORs for dispensed drugs in all categories analyzed, at all time points. The ORs decreased the second years for all drugs analyzed except for propiomazine and stimulants.

**Fig. 2:**
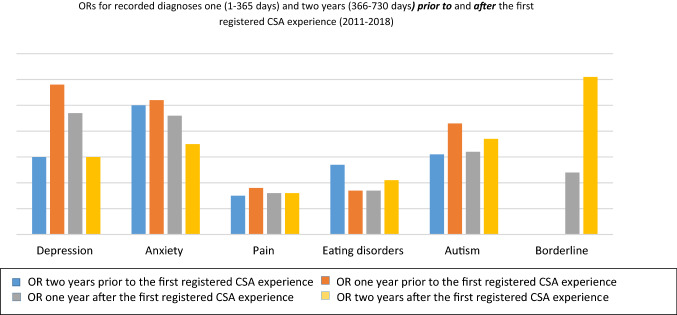
ORs among adolescent girls with a registered CSA experience, as compared to adolescent girls without a registered CSA experience, for diagnoses with a decrease of ORs 1 year after the first registered CSA experience, and with lower (group 1 diagnoses; depression, anxiety, and pain) or higher ORs (group 3 diagnoses; eating disorders, autism, and borderline disorder) the second year *after* the first registered CSA experience. The ORs prior to the first registered CSA experience are collected from a previous study in the same cohort [19]

**Fig. 3:**
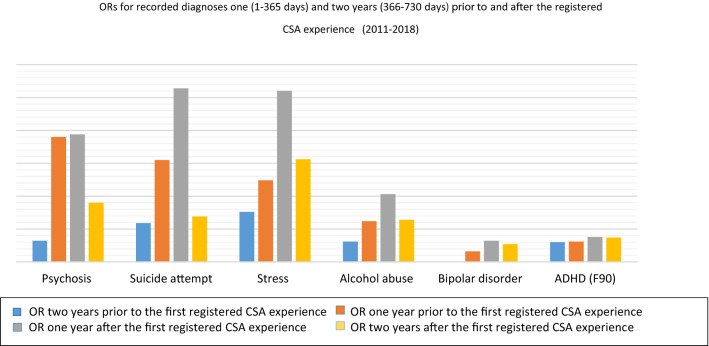
ORs among adolescent girls with a CSA experience registration, as compared to adolescent girls without a CSA experience registration with an increase of OR 1 year after the first registered CSA experience (group 2 diagnoses; psychosis, suicide attempt, stress disorders, alcohol abuse, bipolar disorder, and ADHD) with and with remaining higher OR the second year after the first registered CSA experience, compared to 2 years *before* the first registered CSA experience. The ORs prior to the first registered CSA experience are collected from a previous study in the same cohort [19]

Three different trajectories of diagnoses could be identified supported by the OR development during the period analyzed. In group 1 (depression, anxiety, and pain), the ORs fell to a lower level the year after the first registered CSA experience, and continued to fall the second year after the first registered CSA experience **(**Fig. 2). In group 2 (psychosis, suicide attempts, stress-related diagnoses, alcohol abuse, bipolar disorders, and ADHD), the ORs for the incidence of these diagnoses rose 1 year after the first registered CSA experience and then fell the second year after the first registered CSA experience (Fig. 3). Finally, in group 3 [eating disorders, autism spectrum disorder, and borderline personality disorders (BPD)], the common denominator was a higher OR 2 years after the first registered CSA experience, than the first year after the first registered CSA experience (Fig. 2).

Both number of visits and medications analyzed, except for propiomazine and stimulants, followed the group 2 pattern, with a peak 1 year after the first registered CSA experience. Propiomazine and stimulants instead, followed the group 3 pattern, with an increased usage 2 years after as compared to the usage 1 year after the first registered CSA experience.

## Discussion

The main finding of this cohort study was that the high healthcare consumption levels and the different healthcare consumption pattern among adolescent girls 1 and 2 years prior to their first registered CSA experience remained high and differed from the controls both 1 and 2 years after the first registered CSA experience. The results highlight the complex associations between mental health problems, sexual abuse, and disclosure. The study is observational and conclusions on causality cannot be drawn. Nevertheless, the results support earlier studies suggesting disclosure per se is not enough to lower the psychological burden of CSA experiences, but is rather the start of a vulnerable process [24], with increased risks of suicidal actions.

In summary, the most striking decreases in ORs for symptoms were seen in group 1 (depression, anxiety, and pain). Despite this, ORs for depression and anxiety remained more than three times higher for the girls with a CSA experience registered compared to the controls even 2 years after the first registered CSA experience, and the levels of collection of antidepressants and tranquilizers remained more than five times higher for the girls with a CSA experience registration compared to the controls. However, looking further into the data, we found that the incidence of recorded diagnoses of depression and anxiety among the girls with a CSA experience registration increased by approximately 150% during the same period (not shown in tables), thus indicating that the decrease in ORs for the girls with a CSA experience registration is more likely to reflect a general increase of depression and anxiety among adolescent girls as they grow older, than a decrease of symptoms among the girls with a CSA experience registration. In group 2 (psychosis, suicide attempts, stress-related diagnoses, alcohol abuse, bipolar disorders, and ADHD), an alarming and even dangerous pattern was seen, with ORs of 26 for incidences of stress disorders as well as of suicide attempts 1 year after the first registered CSA experience. An increase of stress before, during, and after disclosing and seeking help for stressful life events is expected and has been seen in prior studies on adults [25]. Suicidal ideation and suicidal attempts have also been strongly associated with CSA in earlier studies [26]. However, the high level of OR for diagnosis of suicide attempts after the first registered CSA experience, in combination with an increased number of healthcare visits and medication, indicates that the healthcare offered to these girls after the first registered CSA experience was not enough, or not enough adapted to their needs. The very high ORs for psychotic disorders in group 1 are also interesting. The association between CSA and psychosis has been shown in different studies, including meta-analyses [27, 28]. The high ORs in this study can, hypothetically, be explained by the difficulties of diagnostically differentiating between dissociative disorders and psychotic symptoms which, however, lies outside the design of this study to analyze [29, 30]. Among the group 3 diagnoses, borderline personality disorders (BPD) were not present as a diagnosis at all prior to the first registered CSA experience in the previous study of the cohort [19], but appear the first year, with a drastic rise the second year, after the first registered CSA experience. In a study from1989 by Herman, van der Kolk and Perry, BPD was associated with severe childhood abuse or neglect starting prior to age 7 in 87% of the participants [31]. Several later studies have confirmed the strong association between BPD and adverse childhood experiences in general and for CSA in particular, as well as for associations between a high burden of child abuse, in particular neglect, and persistent self-destructive behavior [26, 31, 32] [33]. Hence, the rise of ORs for BPD in combination with the persistent self-destructive behavior and stress, as reflected in the high ORs for diagnoses of suicide attempts, alcohol abuse, and stress disorders, indicates a high burden of adverse childhood experiences (ACEs) among the girls with CSA experience registration. In the previous study on the same cohort, the high and particular healthcare consumption pattern among the cohort *prior* to the first registered CSA experience was hypothesized to be due partly to delayed disclosure of CSA and other ACEs [19]. The studies mentioned above and the results from the present study strengthen this hypothesis. Moreover, the results from this study strongly indicate that the visits and eventual interventions offered to the adolescent girls were not enough to help them cope with their suicidal ideations.

## Clinical implications

This study highlights the complexity of CSA [24], and the need to systematically evaluate and develop both assessment of symptoms, treatment planning, and interventions offered to adolescent girls after CSA experience registration. National guidelines would most likely result in a greater support for clinicians and thus their patients.

### Strengths and limitations

As far as we know, this is the largest study of health care consumption after the first registered CSA experience, among girls. The well-defined inclusion criteria and the broad data from all forms of care, and from collected and prescribed drugs, are strengths. Another strength is that the diagnostic data were based on clinical assessments and not self-reported. However, the present study was observational and we cannot draw firm conclusions on causality: We know that mental health problems and emotional distress are risk factors for victimization among youth [34] and the girls in this study had mental health problems already before the CSA experience was first registered [19]. Another limitation is that this study is restricted to information about girls with CSA registered in the health care system, where the date of the registration gives no information about the true date of the abuse. Furthermore, the study is limited by individual’s willingness to disclose their experiences of CSA, which we know is often delayed [35] or low among youth [36], and healthcare staff do not routinely ask young people about abuse experiences in Sweden. The fact that only girls are included in the study and the lack of information about the content of the contacts, such as different types of psychotherapy, are also limitations.

## Conclusion

The contacts and medication offered to adolescent girls in the Stockholm Region, Sweden, after their first registered CSA experience, seem not to be sufficient to prevent an increase of suicidal attempts during the following year, nor does it prevent the development of eating disorders and BPD in the aftermath of CSA. The results show that recognizing health care consumption patterns among adolescent girls at risk for CSA for early intervention and treatment need to be supplemented by increased knowledge about helpful interventions for adolescent girls where disclosure has led to a CSA experience registration within the health care system. Further studies on the cohort, with analyses of different somatic and psychosomatic diagnosis and their possible associations with different healthcare consumption patterns among girls exposed to CSA, prior and after the first registered CSA experience within the healthcare system, would be of great interest as well as a longer follow-up period.

### What this study adds

This study adds important information about the development of suicidal actions, stress, eating disorders, and borderline disorder among adolescent girls, after health care professionals have made the first registration of a CSA experience in their journal record.
